# A configurable streaming spiking neural network accelerator with decoupled pixel–level and output–channel parallelism for automatic modulation classification

**DOI:** 10.3389/fnins.2026.1875437

**Published:** 2026-07-08

**Authors:** Kuilian Yang, Ahmed M. Eltawil, Khaled Nabil Salama

**Affiliations:** Computer, Electrical and Mathematical Science and Engineering (CEMSE) Division, King Abdullah University of Science and Technology (KAUST), Thuwal, Saudi Arabia

**Keywords:** automatic modulation classification, configurable architecture, edge computing, FPGA accelerators, quantized inference, sparse computation, spiking neural networks, streaming dataflow

## Abstract

Streaming spiking neural network (SNN) accelerators are widely adopted on edge platforms for their deterministic, low-latency inference. Realizing their full efficiency, however, requires three properties to hold simultaneously: a router-free streaming dataflow, joint exploitation of temporal and spatial sparsity within that dataflow, and configurable parallelism that can adapt to heterogeneous layers and hardware budgets. Existing streaming SNN accelerators typically achieve at most two of these properties: pixel-level and output-channel parallelism are bound to a single fixed operating point, limiting efficient mapping across heterogeneous layers in automatic modulation classification (AMC) workloads. This paper presents a configurable streaming SNN accelerator that satisfies all three properties by explicitly decoupling pixel-level multiple matrix–vector multiplication (MMV) parallelism from output-channel parallelism. The decoupling is realized within a weight-priority gated one-to-all product (GOAP) dataflow by deterministic offline scheduling, preserving router-free streaming execution while exploiting temporal and spatial sparsity. The proposed architecture is implemented on a Xilinx Virtex-7 field-programmable gate array (FPGA) and evaluated using the RadioML 2016 dataset and a compatible subset of RadioML 2018 under multiple sparsity and quantization settings. Experimental results show that, under comparable end-to-end latency, configurable parallelism enables effective layer-wise latency balancing and substantial hardware-resource savings while sustaining high throughput and classification accuracy. More broadly, the same accelerator description can be retargeted across operating points spanning more than an order of magnitude in hardware cost, providing a key enabler for deploying streaming SNNs at scale across diverse edge hardware platforms.

## 1 Introduction

Edge intelligence in modern wireless systems demands real-time, high-throughput inference under tight power and resource budgets. Automatic modulation classification (AMC), which underpins spectrum monitoring, interference mitigation, and adaptive communication (Huynh et al., 2021), is a representative workload of this kind: it must process incoming RF samples continuously and respond with very low latency, leaving little headroom for cloud offloading or runtime arbitration. Spiking neural networks (SNNs) are well-suited to meeting these constraints. By replacing dense multiply–accumulate operations with sparse, addition-only updates, SNNs naturally exploit temporal sparsity and—when combined with weight pruning and quantization—spatial sparsity as well, yielding the multiplier-free, low-power computation ideal for edge deployment (Zhang J. et al., 2023). Together, these properties make SNN-based AMC a representative real-world application of energy-efficient, low-latency intelligence on the network edge—a setting where the practical deployment of SNNs depends critically on architectural support for diverse hardware budgets.

Among hardware substrates for SNN inference, streaming dataflow accelerators on FPGAs are particularly well-matched to the latency and throughput demands of AMC. By mapping each network layer onto dedicated hardware and pipelining inter-layer execution without global routers or runtime schedulers, streaming designs deliver predictable latency at high throughput. Performance in such designs is no longer constrained by global memory bandwidth or runtime arbitration; the dominant bottleneck shifts to how computation is partitioned across layers—that is, to the execution granularity chosen at design time. Yet most existing streaming SNN accelerators bind this granularity to a single fixed operating point in which pixel-level and output-channel parallelism are coupled and set at design time. Because SNN layers in AMC workloads vary substantially in spatial dimensions, channel counts, and sparsity patterns, a single fixed granularity cannot match all layers simultaneously, leaving compute resources poorly balanced across the pipeline and producing severe inter-layer latency imbalance.

Resolving this rigidity is non-trivial because three properties that together define an efficient streaming SNN accelerator are fundamentally in tension: (i) a router-free streaming dataflow, which underpins the predictable latency and high throughput of streaming designs; (ii) joint exploitation of temporal and spatial sparsity within that dataflow, which is essential for translating SNN sparsity into hardware efficiency but inherently produces unbalanced workloads; and (iii) configurable parallelism that can be retargeted to heterogeneous layers and platforms, which conflicts with the rigid, layer-bound compute structures on which streaming designs rely. To our knowledge, no prior accelerator has simultaneously satisfied all three. Our prior sparsity-aware output-channel dataflow streaming (SAOCDS) design (Yang et al., 2026) satisfies the first two but operates at a single fixed design point implicitly determined by layer dimensions and sparsity. This paper closes the remaining gap. We propose a configurable streaming SNN accelerator that explicitly decouples pixel-level multiple matrix–vector multiplication (MMV) parallelism from output-channel parallelism, where MMV denotes the parallel execution of several matrix–vector multiplications corresponding to different spatial output positions within the same output channel. Building on a gated one-to-all product (GOAP) weight-priority dataflow (Lien and Chang, 2022), we develop deterministic load/store scheduling, inter-layer MMV transfer mechanisms, and output-channel grouping strategies that allow MMV and output-channel parallelism to be configured independently while preserving streaming execution without runtime arbitration and continuing to exploit both temporal and spatial sparsity. The contributions of this work lie at the architectural level; the SNN models, training, and quantization techniques used in our evaluation follow standard practice and are not the main contribution of this paper.

Resolving this rigidity is non-trivial because three properties that together define an efficient streaming SNN accelerator are fundamentally in tension: (i) a router-free streaming dataflow, which underpins the predictable latency and high throughput of streaming designs; (ii) joint exploitation of temporal and spatial sparsity within that dataflow, which is essential for translating SNN sparsity into hardware efficiency but inherently produces unbalanced workloads; and (iii) configurable parallelism that can be retargeted to heterogeneous layers and platforms, which conflicts with the rigid, layer-bound compute structures on which streaming designs rely. To our knowledge, no prior accelerator has simultaneously satisfied all three. Our prior sparsity-aware output-channel dataflow streaming (SAOCDS) design (Yang et al., 2026), built on the gated one-to-all product (GOAP) weight-priority dataflow (Lien and Chang, 2022), satisfies the first two, but its per-layer parallelism is structurally bound, at the algorithm level, to the spatial extent of each output channel: once the network topology is fixed, the parallelism of every streaming stage is fixed with it, and no configuration mechanism is exposed at deployment. This rigidity leaves no room to adapt the same trained model to different hardware budgets or application targets. This paper closes the remaining gap by proposing a configurable streaming SNN accelerator that explicitly decouples output-channel parallelism from pixel-level multiple matrix–vector multiplication (MMV) parallelism, where MMV refers to the parallel execution of several matrix–vector multiplications across different spatial positions within an output channel. The decoupling is realized through deterministic load/store scheduling, inter-layer MMV transfer mechanisms, and output-channel grouping strategies that allow both parallelism dimensions to be configured independently while preserving streaming execution without runtime arbitration and continuing to exploit both temporal and spatial sparsity. As a result, the same architecture can be retargeted across heterogeneous deployment scenarios—from latency-critical to resource-constrained settings—purely through configuration. The contributions of this work lie at the architectural level; the SNN models, training, and quantization techniques used in our evaluation follow standard practice and are not the main contribution of this paper.

The key contributions of this paper are summarized as follows:

1. We propose a configurable streaming SNN accelerator that decouples pixel-level MMV parallelism from output-channel parallelism within a single router-free pipeline. The decoupling is supported within a deterministic weight-priority streaming dataflow by inter-layer MMV transfer, offline output-channel grouping, and precomputed load/store scheduling, all of which preserve fully streaming execution without runtime arbitration. To the best of our knowledge, this is the first streaming SNN accelerator to satisfy all three properties of router-free dataflow, joint temporal–spatial sparsity exploitation, and configurable parallelism simultaneously.
2. Building on this configurability, we develop an inter-layer latency balancing methodology that mitigates the throughput and resource bottlenecks of fixed-granularity streaming designs. Per-layer tuning of MMV and output-channel parallelism aligns latency across heterogeneous layers through architectural configuration rather than manual sparsity tuning, eliminating the need to over-provision hardware for worst-case layers.
3. We provide a comprehensive FPGA evaluation on the Xilinx Virtex-7 platform using the RadioML 2016 dataset and a compatible subset of RadioML 2018 under multiple sparsity and quantization settings (16-bit, 8-bit, and 4-bit), showing that the same accelerator description can be retargeted across operating points spanning more than an order of magnitude in hardware cost while sustaining competitive throughput and classification accuracy.

The remainder of this paper is organized as follows. Section 2 reviews prior work on SNN accelerators, configurable neural network accelerators, and FPGA-based AMC designs. Section 3 presents the proposed configurable streaming architecture, including the supporting algorithms, scheduling mechanisms, and dataflow. Section 4 describes the experimental setup and reports the software and FPGA results. Section 5 discusses the architecture's scalability and the limitations of the present implementation, and Section 6 concludes the paper.

## 2 Related work

General-purpose processors such as GPUs and TPUs are optimized for dense, regular tensor operations and translate poorly to the event-driven, sparse computation that characterizes SNNs, in which most neurons are inactive at any given timestep (Roy et al., 2019). This mismatch has driven the development of dedicated SNN accelerators, ranging from large-scale neuromorphic chips such as TrueNorth (Merolla et al., 2014) and Loihi (Davies et al., 2018) to a growing body of FPGA- and ASIC-based designs. The proposed accelerator builds on three lines of this prior research that have, to date, evolved largely in isolation: streaming SNN accelerators that pursue router-free, fully pipelined inference; sparsity-aware accelerators that exploit temporal and spatial sparsity in SNNs; and configurable accelerators that expose execution granularity as a tunable parameter. We review each in turn before discussing prior FPGA-based AMC accelerators, which constitute the application context of the present work.

### 2.1 Streaming SNN accelerators

Streaming SNN accelerators map each network layer onto dedicated hardware and execute inter-layer pipelines in a router-free manner, eliminating global routers and runtime schedulers. Representative FINN-based designs (Umuroglu et al., 2017; Khodamoradi et al., 2021; Guo et al., 2023) achieve high throughput with an input-priority sliding-window dataflow, but their inability to skip zero weights without runtime control makes it difficult to translate spatial sparsity into proportional hardware savings. DeepFire2 (Aung et al., 2023) pursues a complementary direction, mapping large-scale convolutional SNNs across multiple super logic regions of a multi-die FPGA to improve resource allocation and clock frequency, but it likewise operates at a fixed execution granularity and does not exploit weight sparsity. Our prior work (Yang et al., 2026, 2025) introduced a GOAP-based (Lien and Chang, 2022) weight-priority streaming dataflow that precomputes weight metadata offline so that both temporal and spatial sparsity can be exploited statically without runtime arbitration. The resulting accelerator, however, retains a fixed execution granularity: pixel-level MMV parallelism is bound to the full spatial extent of an output channel, and output-channel parallelism cannot be configured independently.

### 2.2 Sparsity-aware SNN accelerators

A separate line of research targets sparsity exploitation as the primary architectural objective, typically outside the streaming setting. Skydiver (Chen et al., 2022) exploits spatio-temporal sparsity through channel-wise workload prediction and runtime scheduling, and explicitly observes that sparse SNN execution introduces unpredictable and unbalanced workloads—a tension that becomes more acute under the rigid dataflow assumptions of streaming designs. SATO (Liu et al., 2024) pushes this direction further on ASIC, using temporal-unrolled parallelism to accumulate membrane potentials across timesteps in parallel and a bucket-sort-based dispatcher to redistribute compressed sparse workloads across processing elements. More recently, FireFly-S (Li et al., 2024) brings dual-side sparsity exploitation to FPGA through a Bitmap-based sparse decoder and a parametric spatial architecture with inter-layer pipelining, achieving high efficiency on small image-classification benchmarks. These designs demonstrate that sparsity-aware execution can deliver substantial efficiency gains, but they each rely on runtime arbitration, regenerated per-network instances, or dispatcher logic to handle the workload imbalance induced by sparsity, and therefore do not provide the deterministic, router-free dataflow that streaming designs require.

### 2.3 Configurable neural network accelerators on FPGA

A third line of work focuses on making the accelerator itself adaptable to different networks and resource budgets. Choudhury et al. (2022) propose an FPGA overlay for CNNs whose parallelism factors are reconfigured at runtime via host-issued control words, motivated by the observation that streaming dataflows “favor customization over flexibility.” For SNNs, Spiker+ (Carpegna et al., 2024) provides a generation framework in which neuron models, network topology, and per-layer parameters are exposed as design-time knobs, regenerating a customized accelerator for each target network. Both efforts establish configurability as a first-class design objective, but neither preserves a fully streaming, statically scheduled dataflow: the overlay of Choudhury et al. (2022) relies on time-multiplexed reuse and runtime reconfiguration in place of layer-pipelined execution, whereas Spiker+ regenerates a fixed accelerator instance per network rather than tuning execution granularity within a single streaming pipeline. The present work targets exactly this gap, treating configurability as a property of the streaming dataflow itself rather than as an alternative to it.

### 2.4 FPGA-based AMC accelerators

AMC has been targeted on FPGA platforms through several architectural styles that trade flexibility against efficiency in different ways. Early ANN-based designs (Emad et al., 2021; Zhang K. et al., 2023) achieve competitive AMC accuracy on RadioML 2016 through dense MAC operations, but their non-streaming execution incurs high compute cost and limits inter-layer parallelism. Transformer-based AMC accelerators (Wang et al., 2024) pursue richer model capacity at the cost of high latency and noticeable accuracy degradation under reduced precision. More recent non-streaming ANN accelerators (Rose et al., 2025; Kamaleldin et al., 2026) target alternative optimization objectives such as minimal hardware cost and high-throughput programmable execution. The streaming ANN-based design of (Maclellan et al., 2024) combines pipelined inter-layer execution with an RFSoC front-end but remains bounded by its front-end processing stage rather than by its neural-network compute. Streaming SNN-based AMC accelerators have also been explored (Guo et al., 2023; Yang et al., 2026, 2025); their architectural characteristics are discussed in Section 2.1. The quantitative hardware trade-offs of these designs are analyzed in Section 4.6.

Across the four research directions reviewed above, no prior work has combined sparsity-aware streaming execution with configurable parallelism within a single router-free dataflow. The present work is built around the goal of closing this gap, and the architectural mechanisms that achieve it are presented in the next section.

## 3 Proposed configurable streaming architecture

**Figure 1:**
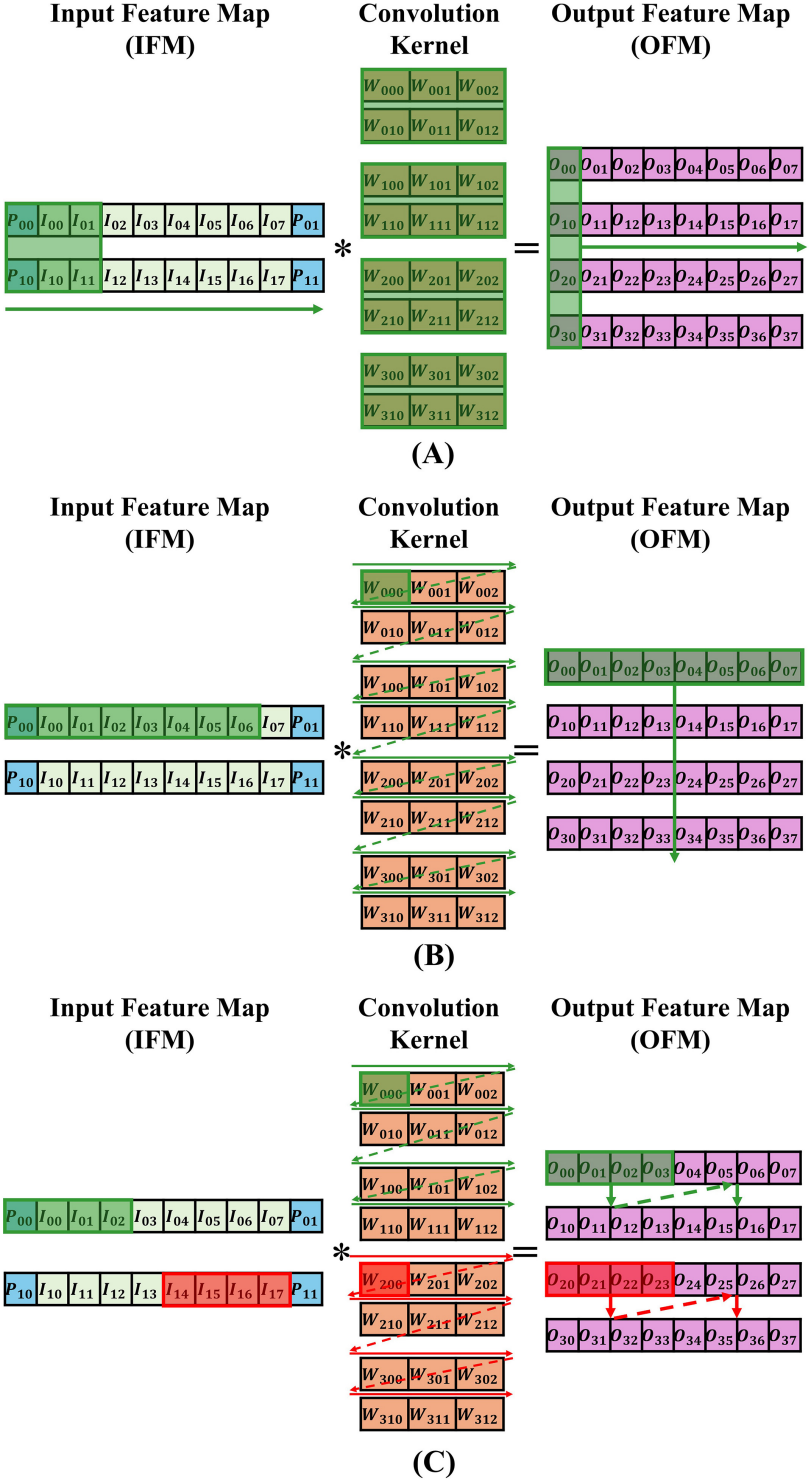
Iteration schemes of three convolution algorithms: **(A)** sliding window (SW) (Guo et al., 2023), **(B)** gated one-to-all product (GOAP) (Yang et al., 2026), and **(C)** GOAP with MMV and output-channel parallelism support. Arrows over the IFM denote input-priority iteration, whereas arrows over the kernel denote weight-priority iteration. Colored boxes without arrows indicate data selected by the iteration order; for the OFM, they indicate the generation sequence. The symbol * denotes the convolution operation.

At a high level, the proposed accelerator computes each convolutional layer through a fixed, layer-pipelined dataflow in which weights are streamed from layer-local memory and partial sums are accumulated as inputs arrive. Configurability enters this dataflow along two independent dimensions. *MMV parallelism* controls how many spatial output positions of one channel are computed concurrently—larger values raise instantaneous throughput at the cost of wider datapaths and more buffering, while smaller values trade peak parallelism for lower per-iteration hardware cost. *Output-channel parallelism* controls how many output channels are processed simultaneously by replicating the compute path across channel groups, trading hardware cost for layer latency. Decoupling these two dimensions is what enables a single accelerator description to be retargeted across heterogeneous layers and hardware budgets. The remainder of this section develops the mechanisms required to make this decoupling realizable within a router-free streaming pipeline: Section 3.2 introduces the MMV padding and inter-layer transfer schemes that support configurable MMV; Section 3.3 addresses the workload balancing and load/store scheduling required for output-channel parallelism; and Section 3.4 integrates both into the overall dataflow. Throughout the section, we use the running convolution example shown in Figure 1 to illustrate the resulting dataflow behavior, with dimensions defined as follows:

- Input feature map (IFM): (1, 10, 2), representing height (*H*), width (*W*), and number of input channels (*IC*), respectively.
- Kernel: (1, 3, 2, 4), corresponding to *H*, *W*, *IC*, and number of output channels (*OC*).
- Output feature map (OFM): (1, 8, 4), denoting *H*, *W*, and *OC*.

Because radio frequency (RF) signals are processed as one-dimensional sequences per channel, only horizontal convolution is applied, and we assume a convolution stride of one with symmetric padding of one on both sides. In the IFM, green cells denote input data received from the preceding layer, whereas blue cells indicate the padding applied to maintain the desired OFM dimensions. Figure 1 introduces three convolution iteration schemes under this setting, which the next subsection analyzes in detail.

### 3.1 Limitations of coupled parallelism in prior streaming dataflows

The input-priority sliding window (SW) dataflow of Figure 1A (Guo et al., 2023) produces, at each iteration, one spatial output position simultaneously across multiple output channels. Parallelism is therefore exposed exclusively along the output-channel axis and capped by the layer's output-channel count—four in the running example. No mechanism exists to additionally parallelize across spatial positions within a channel, so layers with few output channels but wide spatial extents lock in the pipeline-wide minimum latency at the algorithm level, with no further acceleration possible regardless of available hardware.

The weight-priority GOAP dataflow of Figure 1B, adopted in our prior work (Yang et al., 2026), shifts the binding to the opposite axis: each iteration broadcasts a single weight across all spatial positions of one output channel, so parallelism is pinned to the channel's spatial extent—eight in the running example—while output channels are processed strictly one at a time. The result is a single, fully determined operating point per layer: layers with long output channels cannot scale parallelism down to relieve hardware pressure, while layers with many short output channels cannot scale up by processing several channels concurrently.

The proposed dataflow of Figure 1C decouples these two axes. MMV can be set to any divisor of the channel's spatial extent and output-channel parallelism to any divisor of the channel count, with total parallelism ranging from a single pixel up to the full OFM. Moreover, the same degree can be realized in qualitatively different ways: in the running example, a degree of eight admits 1 × 8, 2 × 4, and 4 × 2 partitions of (output channels) × (spatial positions per channel), each with distinct buffering, scheduling, and resource implications. Parallelism becomes a design-time knob rather than a fixed property of the layer, allowing each layer to be tuned for the target network topology, hardware budget, and deployment scenario.

### 3.2 MMV parallelism support in the algorithm

Configuring MMV parallelism independently of output-channel parallelism requires algorithm-level support for two scenarios: padding block boundaries correctly when MMV is smaller than the channel's spatial extent, and regrouping partial outputs across layers that operate at different MMV values. We address these in turn, then characterize the resulting configuration trade-offs.

#### 3.2.1 MMV padding scheme

**Figure 2:**
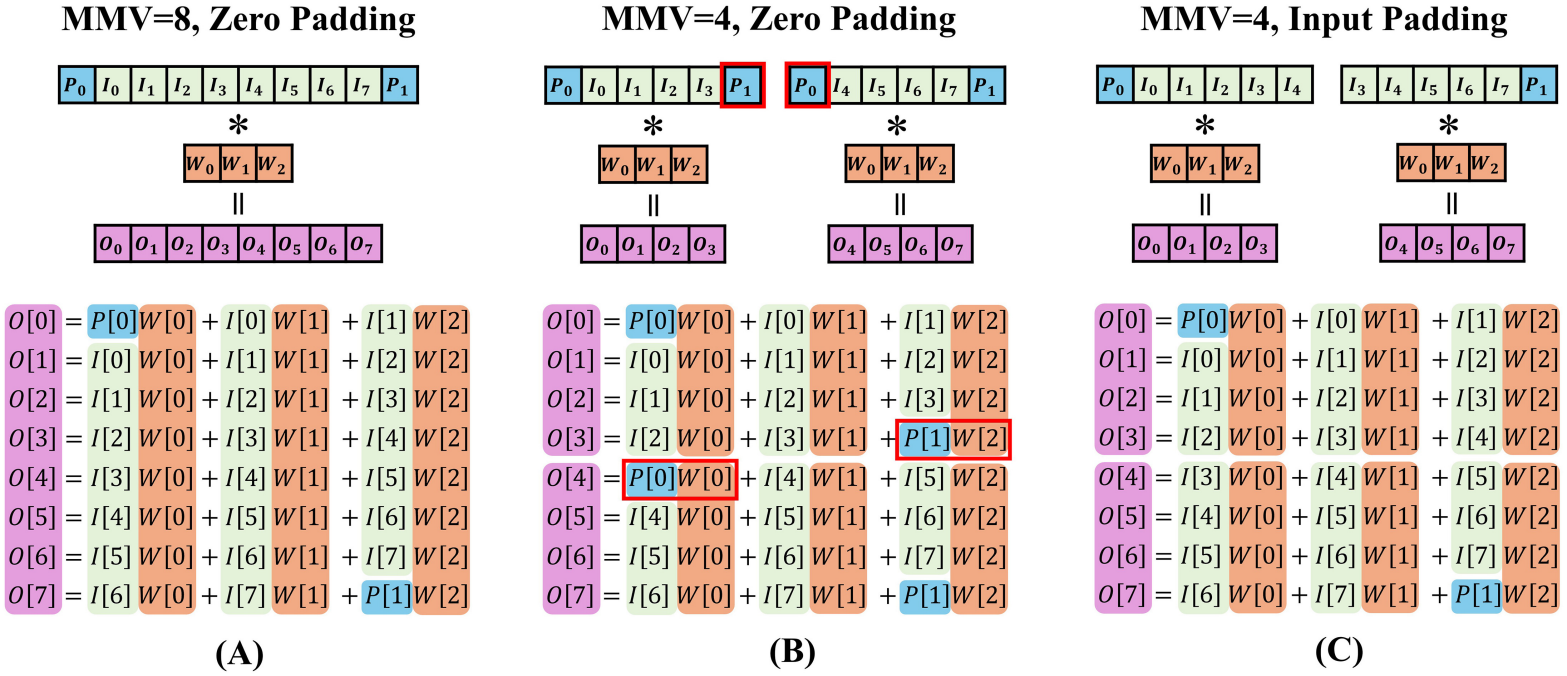
Comparison of IFM padding schemes: **(A)** conventional full-channel padding, **(B)** MMV padding with zeros, and **(C)** MMV padding with input values. The symbol * denotes the convolution operation.

When MMV spans the entire output channel, the channel is processed as a single block with *P*_0_ and *P*_1_ at its two ends, as shown in Figure 2A; all required inputs are then locally available and no special handling is needed.

When MMV instead covers only a portion of the channel, the channel is divided into multiple blocks, each producing MMV outputs in parallel. Outputs near a block boundary depend on inputs that physically reside in the adjacent block. Naively reusing the channel-end padding values *P*_0_ and *P*_1_ at every block boundary, as in Figure 2B, severs these dependencies: *O*_3_ and *O*_4_ should consume *I*_4_ and *I*_3_, respectively, but instead read the padding values *P*_1_ and *P*_0_, producing results inconsistent with the full-channel computation. The proposed scheme, illustrated in Figure 2C, pads each block with the boundary inputs of its neighbor—the left block extends rightward with *I*_3_, *I*_4_, and the right block extends leftward with *I*_3_—restoring the original outputs while retaining block-level parallelism.

#### 3.2.2 Inter-layer MMV transfer and padding

The padding scheme above handles within-layer block boundaries, but different layers may adopt different MMV values to balance their workloads, so inter-layer transfer must additionally support boundary padding across mismatched MMV sizes. To keep the dataflow uniform across all *MMV*_0_/*MMV*_1_ ratios, padding is performed at a fixed granularity called the *block*, with size max(*MMV*_0_, *MMV*_1_). The padding for each block is completed only after the next block has been fully received from layer 0, so that the borrowed boundary input is always available regardless of how either layer iterates internally. We further constrain *MMV*_0_ and *MMV*_1_ to be integer multiples of each other, ensuring that block boundaries align with both layers' iteration boundaries.

**Figure 3:**
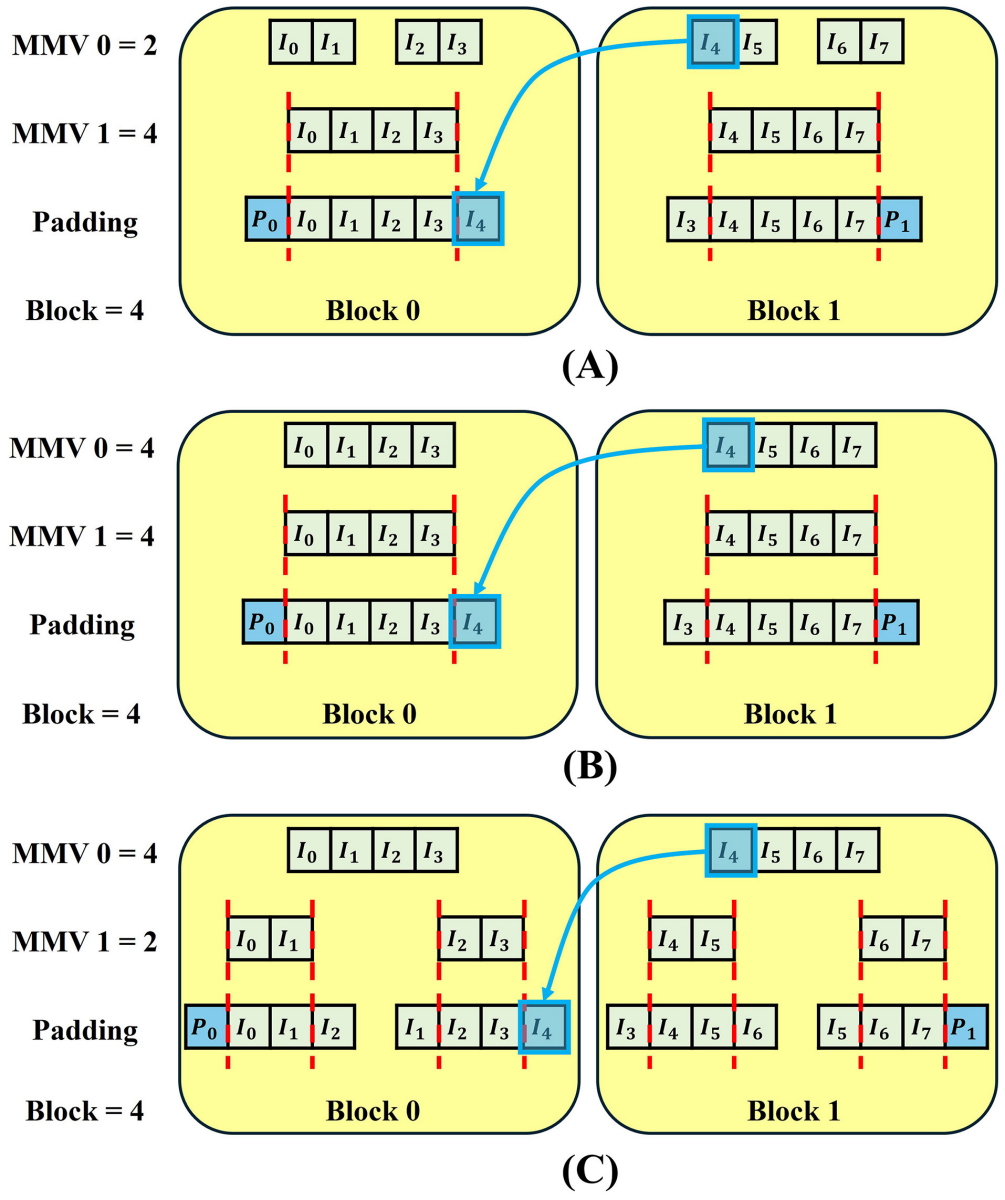
Inter-layer MMV length transfer and padding mechanism under different conditions: **(A)** *MMV*_0_<*MMV*_1_, **(B)** *MMV*_0_ = *MMV*_1_, and **(C)** *MMV*_0_>*MMV*_1_.

Figure 3 illustrates the three resulting cases. When *MMV*_0_ = *MMV*_1_ (Figure 3B), each layer-0 output corresponds to one layer-1 block. When *MMV*_0_<*MMV*_1_ (Figure 3A), layer 1 assembles *MMV*_1_/*MMV*_0_ consecutive layer-0 outputs into one block before padding. When *MMV*_0_>*MMV*_1_ (Figure 3C), each layer-0 output is unpacked into *MMV*_0_/*MMV*_1_ smaller MMV blocks padded in sequence. In all three cases, the unified rule—“pad the previous block when the next block arrives”—introduces at most one iteration of delay per block, which is absorbed by the layer-pipelined streaming architecture and does not accumulate across layers.

#### 3.2.3 MMV configuration trade-offs

**Figure 4:**
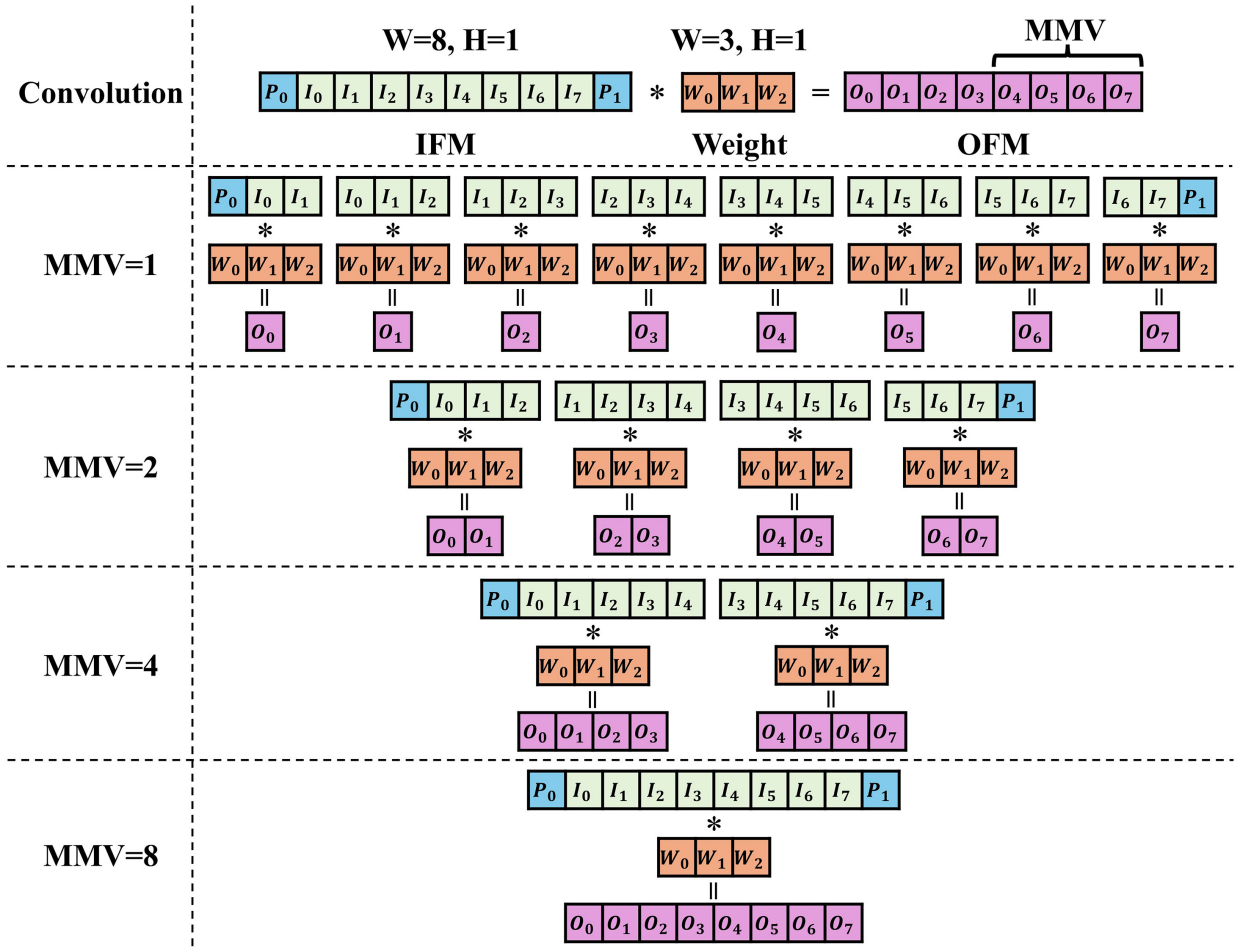
Overhead of IFM fetches under different MMV configurations. The symbol * denotes the convolution operation.

Configuring MMV introduces trade-offs among parallelism, iteration count, and buffering overhead. Larger MMV values increase the number of concurrently computed pixels and reduce the iteration count, but require wider datapaths and higher per-iteration hardware resources; smaller MMV values invert this trade-off. As Figure 4 illustrates, smaller MMV values also incur higher IFM fetch overhead, since each partial-channel block requires its own boundary inputs and the same input position is read by multiple adjacent blocks.

When both temporal and spatial sparsity are exploited under the GOAP-based dataflow (Yang et al., 2026), however, the total number of input fetches and accumulations remains approximately constant across MMV configurations, since the underlying workload does not change (Table 1). The primary effect of MMV scaling is therefore a redistribution of computation across time and hardware resources, motivating the use of MMV as a configurable architectural parameter rather than a fixed design choice.

**Table 1:** Comparison between the SW method (Guo et al., 2023) and four MMV configurations under the GOAP dataflow in terms of the number of weight fetches, input fetches, accumulations, parallel pixel computations, and buffered input data.

Design	# Weight fetch	# Input fetch	# Accumulation	# Pixels computing in parallel	#Buffered input data
SW	3 × 8 = 24	3 × 8 = 24	24·*ts*	3	(1+2) × 8 = 24
MMV = 8	3 × *ss* = 3·*ss*	3 × *ss*×8 = 24·*ss*	24·*ss*·*ts*	8	8+2 = 10
MMV = 4	3 × *ss*×2 = 6·*ss*	6 × *ss*×4 = 24·*ss*	24·*ss*·*ts*	4	(4+2) × 2 = 12
MMV = 2	3 × *ss*×4 = 12·*ss*	12 × *ss*×2 = 24·*ss*	24·*ss*·*ts*	2	(2+2) × 4 = 16
MMV = 1	3 × *ss*×8 = 24·*ss*	24 × *ss*×1 = 24·*ss*	24·*ss*·*ts*	1	(1+2) × 8 = 24

### 3.3 Output-channel parallelism support in the algorithm

The previous subsection described how MMV parallelism is supported within the GOAP-based streaming dataflow. To further enhance the adaptability of the design, we now introduce parallelism across multiple output channels. This subsection discusses the challenges of output-channel parallelism, presents the corresponding load/store strategy, and analyzes the associated overhead.

#### 3.3.1 Workload balancing under output-channel parallelism

Supporting multi-output-channel execution involves more than replicating single-channel hardware: it introduces new load imbalance issues. In single-channel designs (Yang et al., 2026), output channels are processed sequentially, eliminating the need for workload balancing. When multiple channels are computed concurrently, however, spatial sparsity causes the number of non-zero weights per kernel to vary, leading to unequal computation times and idle cycles for faster channels.

**Figure 5:**
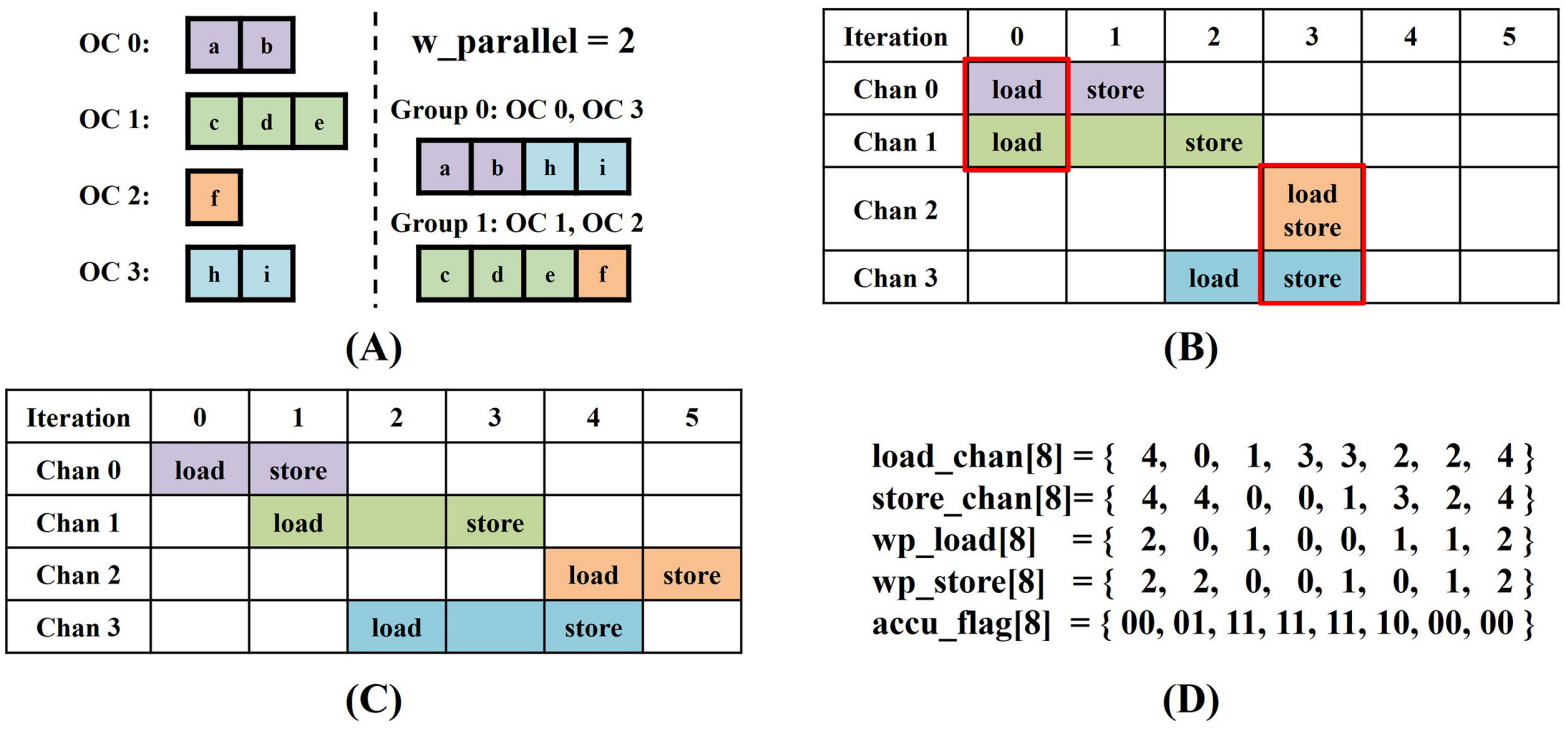
Workload balancing and load/store scheduling for output channel groups. **(A)** Output channels are partitioned into groups assigned to two WPs to balance the total weight iterations per WP. **(B)** A naive schedule allowing multiple loads and stores per iteration leads to access contention on the shared state buffer. **(C)** Under the proposed one-load/one-store policy, two priority rules yield a deterministic load/store order with no runtime arbitration. **(D)** The resulting per-iteration control sequences (*load*_*chan*, *store*_*chan*, *wp*_*load*, *wp*_*store*, *accu*_*flag*) that the hardware follows at runtime.

We define *w*_*parallel* as the number of output channels computed in parallel. Each output channel is assigned to a dedicated hardware unit, denoted WP, which performs the accumulation and state-update operations for its assigned channels. When *w*_*parallel* is smaller than the total number of output channels, each WP handles multiple channels, and spatial sparsity causes the number of non-zero weights per WP to vary, leading to workload imbalance across WPs. This imbalance can be mitigated by reorganizing the channel groups so that the total number of weight iterations per WP is more uniform. Figure 5A illustrates this on a four-channel layer with *w*_*parallel* = 2: the channels are partitioned into two balanced groups, each processed by one WP.

Because weight values and their distributions are fixed during inference, grouping can be determined offline by enumeration or heuristic algorithms. For modest problem sizes, exhaustive enumeration finds optimal groupings; for larger workloads, low-complexity heuristics such as the longest processing time (LPT) heuristic and MultiFit bin-packing provide practical solutions with bounded computation time (Graham, 1969; Chung et al., 2019). Weight data are stored in the COO format as in Yang et al. (2026) and concatenated according to the grouping results, with the weights of each group assigned to the same WP to maximize load balance.

#### 3.3.2 Load/store strategy

##### 3.3.2.1 Load/store scheduling policy

While grouping addresses workload imbalance across WPs, a separate challenge remains: contention on the global state buffer. Each iteration consists of three ordered stages—load, accumulate, and store—and allowing multiple channels to access the buffer simultaneously, as in Figure 5B, would require selecting among several state buffer blocks within a single iteration. This in turn would inflate the control logic and critical path, lowering the achievable operating frequency. We therefore enforce a strict policy in which each iteration allows at most one load and at most one store. Since all weights and their metadata are fixed during inference, the corresponding load/store order can be precomputed offline, with the hardware simply following the resulting control sequences at runtime.

The policy is realized through four iteration-indexed control variables, generated offline. The variables *load*_*chan* and *store*_*chan* identify the output channel whose state is loaded or stored in the current iteration; *wp*_*load* and *wp*_*store* identify the corresponding source or destination WP, each of which maintains a local state buffer for the channels it currently operates on. Most iterations require neither a load nor a store, so each variable is compared with its previous value: a change triggers the corresponding hardware operation, and an unchanged value skips it. This comparison-based scheme allows the four variables to operate independently. A separate multi-bit signal *accu*_*flag* enables or disables accumulation per WP (1 bit per WP).

When multiple WPs compete for a load or store slot in the same iteration, contention is resolved during offline sequence generation by two priority rules: WPs with more remaining non-zero weights are served first, with ties broken by WP index. Applying these rules to the workload of Figure 5A yields the iteration timing diagram in Figure 5C and the corresponding precomputed control sequences in Figure 5D. The complete computation requires six effective iterations, while all control sequences have length eight: the additional two iterations correspond to reading the IFM from the previous layer and writing the OFM to the next, and also serve as sentinel values. Because the priority rules are applied entirely offline, the runtime hardware contains no arbiter or scheduler—it simply compares each control variable with its previous value, preserving the fixed dataflow required by the streaming pipeline.

##### 3.3.2.2 Handling of empty and extra iterations

The prior SAOCDS design (Yang et al., 2026) accounts for two special iteration cases—empty and extra iterations—by precomputing them into the fixed streaming dataflow to avoid logical hazards arising from sparsity and strict pipelining. These concerns remain in the multi-output-channel context but are handled differently because of the precomputed load/store scheduling.

Empty iterations occur when a weight requires an IFM value that has not yet been read from the previous layer, typically at the start of a layer. In sequential single-channel execution, this mismatch necessitates an additional idle iteration to avoid incorrect computation. In our multi-output-channel design, computed channels are not propagated to the next layer immediately upon completion; instead, all computed channels are buffered until the layer finishes. Because all inputs for the next layer are therefore acquired before its computation begins, weight traversal can never outpace IFM availability, eliminating this class of hazard entirely. Although buffering introduces some latency, it greatly simplifies inter-layer control and does not accumulate in a fully pipelined flow, making its performance impact negligible.

Extra iterations address cases in which some output channels have few or no non-zero weights, which typically occurs in layers with small kernels and high sparsity. This issue persists under multi-output-channel execution but can be managed through load/store control. During channel grouping, output channels with no non-zero weights are scheduled to avoid conflicts, and because multi-output-channel execution decouples computation order from channel indices, load/store operations for such “empty” channels can be placed at the end of the sequence. If residual imbalance among WPs persists, an additional iteration is appended to the sequences of the faster WPs; this iteration performs only load and store operations and reproduces the effect of the original extra iteration. Because other WPs with more work naturally overlap with it, its impact is largely hidden by pipelined execution.

#### 3.3.3 Overhead of output-channel parallelism

Output-channel parallelism improves inference speed and throughput, but it incurs overheads from two sources: (i) the additional storage of control variables required to coordinate multi-output-channel load/store sequences, and (ii) the potential performance impact of enforcing one load and one store per iteration.

Table 2 compares the control-variable and load/store overheads of the second convolutional layer between the original SAOCDS (Yang et al., 2026) and the proposed multi-output-channel design. Note that the row labeled *w*_*parallel* = 1 corresponds to the SAOCDS single-channel logic and therefore does not introduce additional control variables; it is included only as a reference. This layer contains 5,632 weights with 50% spatial sparsity, and each weight requires 29 bits in COO format (Yang et al., 2026), yielding a total of 81,664 bits of weight storage.

**Table 2:** Comparison of control and load/store overheads associated with output-channel parallelism in the second layer.

Design	Total bit of COO data	Bit of load_chan	Bit of store_chan	Bit of wp_load	Bit of wp_store	Bit of accu_flag	Total bit of control variables	Total bit of control overhead	Overhead percentage	Theoretical number of iterations	NNZ_max	Ite_length
SAOCDS Yang et al. (2026)	81,664	0	0	0	0	0	0	0	0.00%	2,816	2,816	2,816
w_parallel=1	81,664	5	5	0	0	1	11	30,998	37.96%	2,816	2,816	2,818
w_parallel=2	81,664	5	5	1	1	2	14	19,824	24.28%	1,408	1,414	1,416
w_parallel=4	81,664	5	5	2	2	4	18	12,816	15.69%	704	710	712
w_parallel=8	81,664	5	5	3	3	8	24	8,736	10.70%	352	362	364
w_parallel=16	81,664	5	5	4	4	16	34	6,732	8.24%	176	184	198
w_parallel=32	81,664	5	5	5	5	32	52	6,708	8.21%	88	118	129

##### 3.3.3.1 Control variable overhead

The first overhead source is the storage of the control variables *load*_*chan*, *store*_*chan*, *wp*_*load*, *wp*_*store*, and *accu*_*flag*. The bit widths of *load*_*chan* and *store*_*chan* depend on the number of channels (32), requiring five bits each. The bit widths of *wp*_*load* and *wp*_*store* depend on *w*_*parallel*, while *accu*_*flag* scales with the number of WPs, with one bit per WP. The total bits per iteration listed in the “Total Bits of Control Variables” column are then multiplied by *Ite*_*length* to obtain the total control-variable storage, and normalizing this by the COO weight storage yields the overhead ratio.

As shown in Table 2, although the per-iteration control bits increase with output-channel parallelism, the overall overhead decreases because the iteration count drops faster. Even with modest parallelism (*w*_*parallel* = 2), throughput nearly doubles and latency is reduced with less than a 25% increase in control-variable storage. At higher parallelism, this overhead ratio continues to decline, making it acceptable given the substantial performance gains. This trend extends to larger layers as well. Aggregating the per-iteration bits of the five control arrays defined above and normalizing by the COO weight storage gives a closed-form expression for the overhead ratio in Equation 1:


$$\begin{array}{l} \frac{S_{\text{ctrl}}}{S_{\text{weight}}}\approx \frac{2\left\lceil \log_{2}N\right\rceil +2\left\lceil \log_{2}w\text{\_}parallel\right\rceil +w\text{\_}parallel}{w\text{\_}parallel\times \left(16+\left\lceil \log_{2}\left(MN\right)\right\rceil +\left\lceil \log_{2}\left(XY\right)\right\rceil \right)}, \end{array}$$ (1)

where *N* is the number of output channels, *M* the number of input channels, and (*X, Y*) the kernel dimensions. Because *S*_ctrl_ and *S*_weight_ are both proportional to the number of non-zero weights, NNZ cancels in the ratio, so the ratio is independent of the sparsity level. The dependence on layer dimensions enters only through

logarithms of *N*, *MN*, and *XY*: when *N* grows, the numerator and denominator both grow as log_2_*N* and the ratio approaches a finite limit of order 1/*w*_*parallel*; when *M*, *X*, or *Y* grows, only the denominator grows and the ratio decreases. The ratio therefore remains bounded as the layer scales in any direction, and the control-memory overhead does not become an obstacle on substantially larger layers.

##### 3.3.3.2 Performance overhead of load/store sequencing

A second overhead arises because limiting each iteration to one load and one store, while effective at avoiding access contention, prevents reaching the theoretical minimum iteration count. Table 2 shows that, in SAOCDS, the total iteration count equals the number of non-zero weights (2,816). For multi-output-channel configurations, the theoretical iteration count represents the minimum number of iterations required under ideal load balancing without contention.

We define *NNZ*_*max* as the largest number of non-zero weights assigned to any WP after load balancing. For *w*_*parallel* < 16, load balancing is effective, and the actual iteration count *Ite*_*length* equals *NNZ*_*max*+2, where the two extra iterations account for IFM input and OFM output. Under these conditions, contention is masked by the inherent load imbalance, since the most loaded WP performs accumulations in every iteration.

When *w*_*parallel*≥16, increasing the number of WPs intensifies contention. Excluding the first and last iterations, the configurations with *w*_*parallel* = 16 and *w*_*parallel* = 32 require 12 and nine extra iterations, respectively, due to unresolved contention. Even at high parallelism, the relative increase remains modest (below approximately 6%–7%), demonstrating that the proposed load/store strategy effectively mitigates contention while retaining most of the benefit of increased parallelism.

### 3.4 Dataflow design

**Figure 6:**
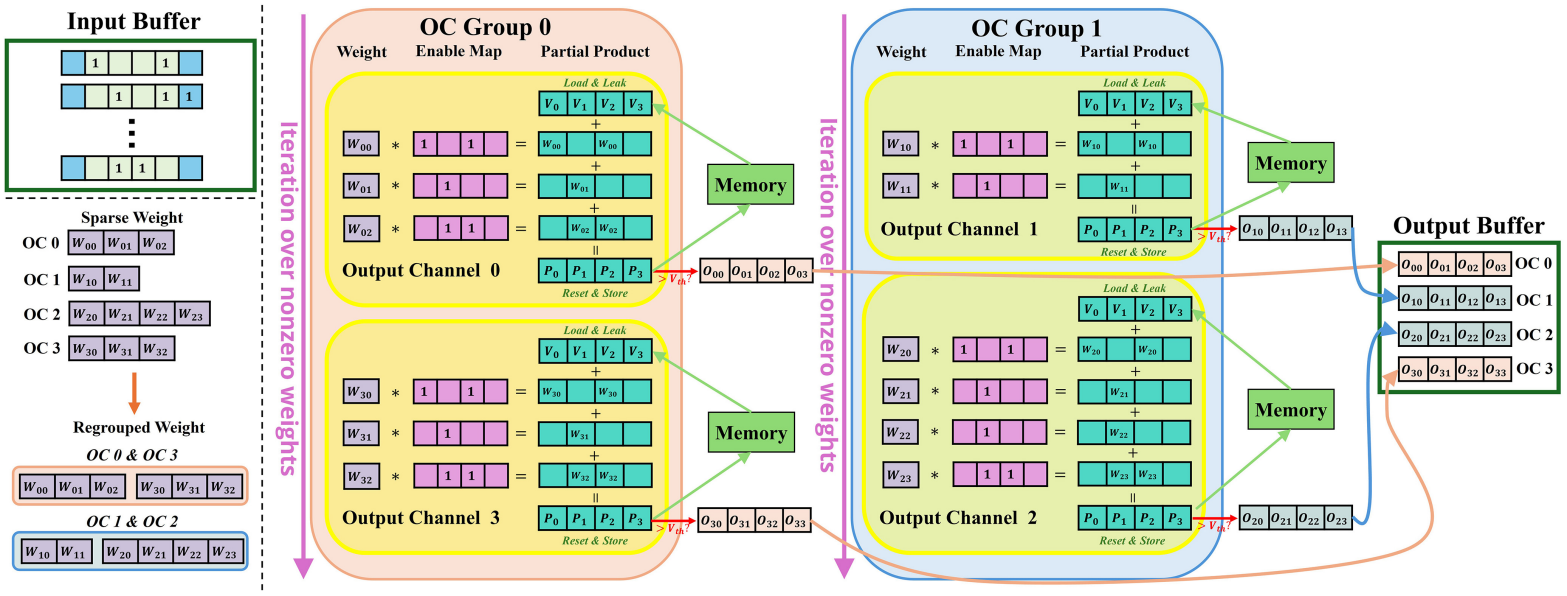
Dataflow for a convolutional layer with configurable MMV and output-channel parallelism. The symbol * denotes the convolution operation.

Once all control variables have been precomputed offline, the dataflow of the streaming accelerator can be arranged. This arrangement is crucial to ensure smooth data transfer within and across layers, correct state storage and retrieval for each WP, and consumption of all non-zero weights in the intended order—without dynamic routers or schedulers. For clarity, Figure 6 illustrates the dataflow of a convolutional layer with *w*_*parallel* = 2.

Following the inter-layer MMV padding scheme, the padded IFM data required to compute the MMV blocks of all channels are first stored in the input buffer. The corresponding weights are grouped and evenly distributed between the two WPs. Each WP then processes its assigned workload by iterating over its non-zero weights. In each iteration, a WP performs the accumulation associated with a non-zero weight and its enable map, and executes state load/store operations at the appropriate times to ensure correctness. Upon completion, each WP produces OFM data for its channel group; once all channels are ready, the OFM—organized in MMV format—is transmitted to the next layer. The complete sequence of operations for generating an MMV block in a single layer is presented in Algorithm 1.

Algorithm 1 Step-by-step pseudo-code for generating an MMV block output in one timestep of a convolutional layer. 
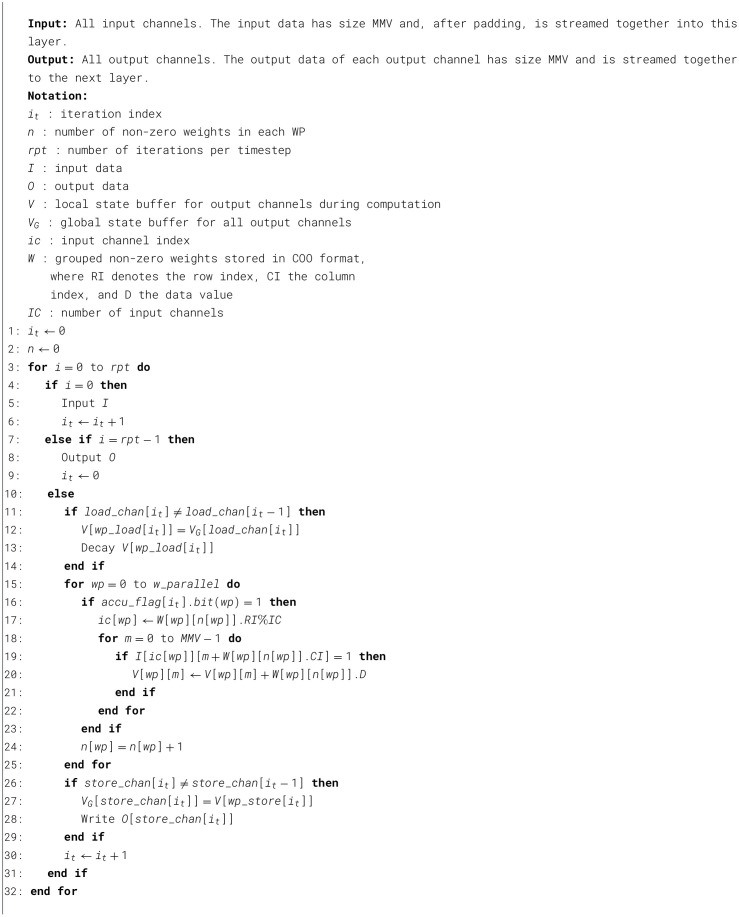

The total number of iterations in Algorithm 1 is fixed by the precomputed control-variable arrays. The first and last iterations transfer the input and output MMV blocks across layer boundaries (lines 4–9), while each intermediate iteration follows the precomputed schedule: *load*_*chan* and *store*_*chan* trigger at most one state load and one state store on the global buffer (lines 11–14, 26–29), and accumulation across all WPs is gated independently by the corresponding bits of *accu*_*flag* (lines 15–25).

Within this single-load/single-store policy, accumulation operations are performed in parallel across all WPs, maximizing parallel computation on the accelerator without requiring additional input/output ports on the state buffer.

## 4 Experimental setup and results

This section evaluates the proposed configurable streaming SNN accelerator. We first describe the datasets and SNN workload used to validate the architecture and report software classification results that establish an algorithmic reference, and then present the FPGA implementation results, including the impact of quantization, an analysis of representative configurable design variants, and a comparison with prior FPGA-based AMC accelerators.

### 4.1 Datasets and SNN workload

#### 4.1.1 Datasets

The proposed design is evaluated on two widely adopted benchmarks: RadioML 2016.10A (O'Shea and West, 2016) and RadioML 2018.01A (O'Shea et al., 2018). RadioML 2016.10A is synthesized through GNU Radio and comprises 11 modulation schemes (eight digital and three analog) over an SNR range of −20dB to +18dB in 2dB steps, with each sample represented as an in-phase and quadrature (I/Q) matrix of size 2 × 128. RadioML 2018.01A contains 24 modulation types over a broader SNR range, with each record consisting of 1,024 I/Q samples. To enable a direct comparison between the two benchmarks, we restrict RadioML 2018.01A to the same 11 modulation classes and SNR range as RadioML 2016.10A. For both benchmarks, raw I/Q samples are converted to binary spike trains using a Sigma–Delta encoding scheme prior to SNN processing, as described in (Guo et al., 2023).

#### 4.1.2 SNN model, pruning, and quantization

**Figure 7:**
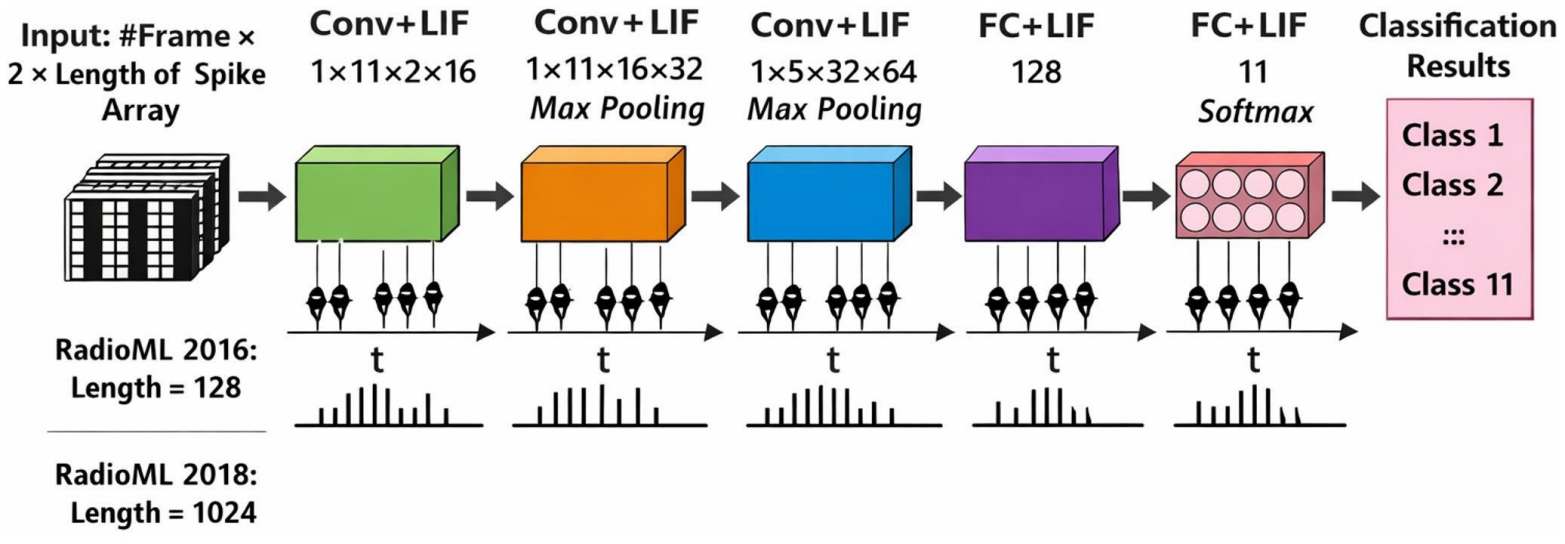
Architecture of the SNN classifier used for both RadioML 2016 and RadioML 2018. *Conv*, convolutional layer; *FC*, fully connected layer; *LIF*, leaky integrate-and-fire neuron. For each convolutional layer, the number beneath denotes the filter dimensions: *kernel*_*y*×*kernel*_*x*×*#input*_*channel*×*#output*_*channel*.

The SNN classifier architecture, adopted from Guo et al. (2021, 2023) as a representative AMC workload, is illustrated in Figure 7. To reduce model complexity while preserving classification performance, we apply fine-grained unstructured pruning Han et al. (2015), in which individual weights are removed based on their *L*_1_ magnitude without altering the network topology. Pruning is integrated into a 100-epoch training schedule for both datasets: the first 20% of epochs are used for feature learning, the next 60% for progressive pruning, and the final 20% for fine-tuning. This schedule yields effective model compression with minimal accuracy loss. In addition, we apply Learned Step Size Quantization (LSQ) (Esser et al., 2019) to obtain low-precision weight representations suitable for hardware inference. LSQ generates 16-bit, 8-bit, and 4-bit fixed-point weights during training, and the resulting quantized weights are used directly for integer-based inference on hardware, enabling a systematic evaluation of the trade-off between model accuracy and hardware efficiency under different precision levels.

### 4.2 Software classification results

**Figure 8:**
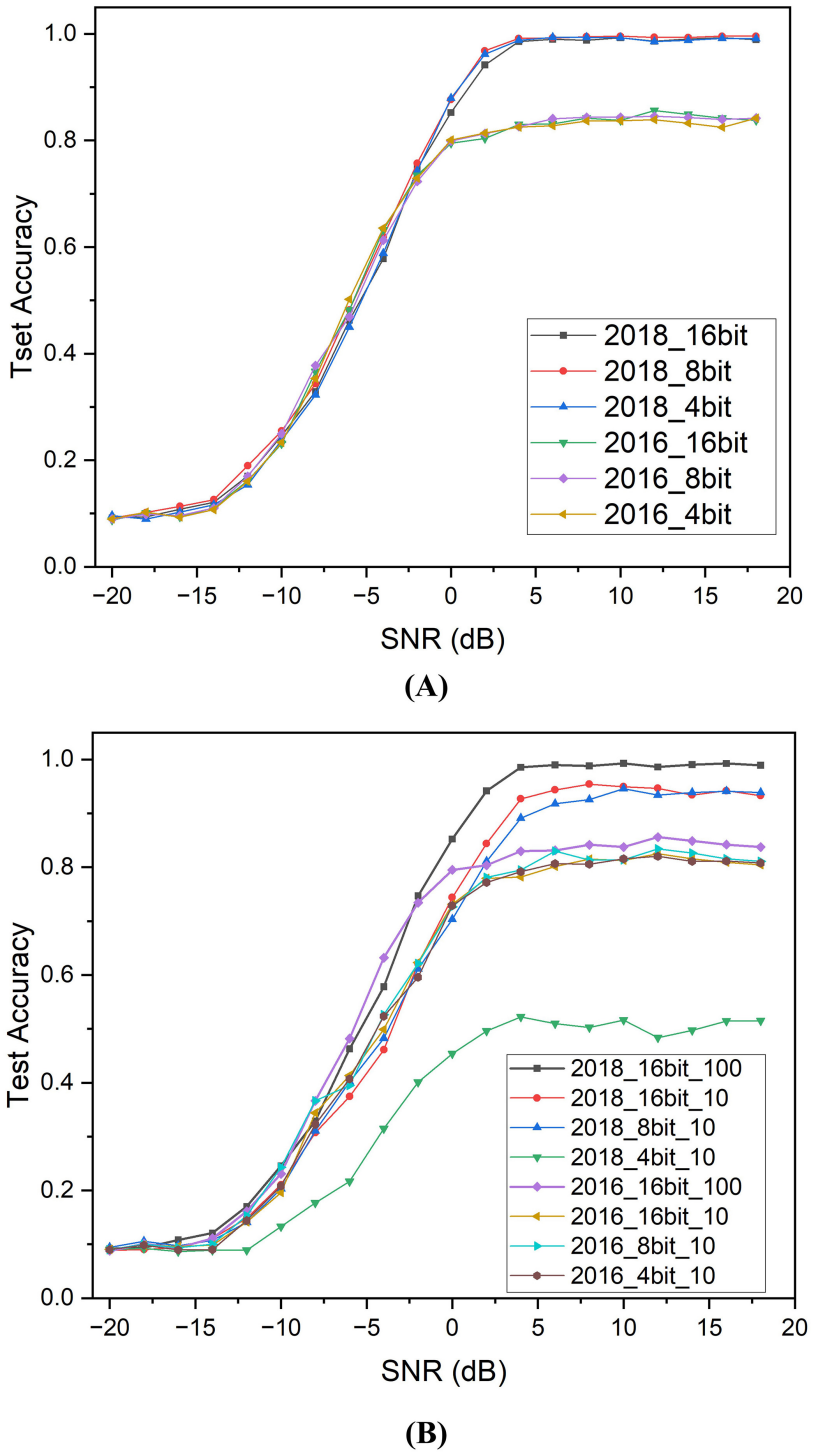
Classification accuracy vs. SNR on RadioML 2016 and RadioML 2018 across quantization bit widths. **(A)** Full-density model. **(B)** Models pruned to a uniform global density of 10%, with a 16-bit unpruned baseline included for reference.

Figure 8 reports the PyTorch classification accuracy under different pruning densities and quantization bit widths, averaged over five independent training runs with different random seeds. Below approximately −15dB, accuracy remains close to random chance for all bit widths, so the analysis below focuses on the SNR regime in which quantization effects are observable. Across the evaluated configurations, the standard deviation of the mean accuracy is below 0.27% in nearly all cases (typically 0.04%–0.21%), with the corresponding 95% confidence intervals spanning only a fraction of a percentage point, indicating that the reported accuracy values are reproducible across runs. The standard deviation rises noticeably only in extreme configurations where the classifier has essentially failed to converge and accuracy itself has already dropped well below the usable range, such as the 2018_4bit_10 curve shown in Figure 8B.

At full density (Figure 8A), accuracy on RadioML 2016 decreases slightly as the bit width is reduced, whereas the curves on the 2018 subset are nearly indistinguishable across precisions. The 2018 subset also reaches markedly higher peak accuracy: for SNR >5dB, RadioML 2016 reaches roughly 80% while the 2018 subset approaches 99%.

Under aggressive uniform pruning at 10% global density (Figure 8B), the effect of quantization becomes more pronounced on the 2018 subset: 16-bit and 8-bit models still perform well, while the 4-bit model shows a clear drop and can fall below the corresponding 2016 result. The 2016 subset is less sensitive in this regime, with differences of only 3%–4% across bit widths for SNR >5dB.

Overall, moderate quantization can be combined with pruning at limited accuracy loss, whereas the most aggressive low-bit setting becomes accuracy-sensitive in this regime. The hardware evaluation in the following subsections therefore adopts a moderate 50% sparsity as the primary operating point, where the efficiency gains of sparsity-aware execution are clearly visible without incurring noticeable accuracy degradation.

### 4.3 FPGA implementation setup

To evaluate the hardware performance of the proposed configurable streaming SNN accelerator, we synthesized and implemented the design on a Xilinx Virtex-7 VC709 FPGA board. The accelerator was described in C/C++ and synthesized to RTL using Xilinx Vitis HLS, followed by integration, placement, and routing in Vivado (Xilinx Inc., San Jose, CA, USA), both at version 2020.2.

On the VC709 platform, we deployed the five-layer SNN classifier shown in Figure 7. Reported hardware costs include LUTs, flip-flops (FFs), block RAMs (BRAMs), and DSPs. Both RadioML 2016.10A and the corresponding subset of RadioML 2018.01A are evaluated under identical quantization settings to assess performance across precision levels.

To quantify overall hardware efficiency, we define a figure of merit (FoM) as shown in Equation 2:


$$\begin{array}{l} \text{FoM}=\frac{\text{Throughput}}{\#\text{LUT}\times \text{Dynamic Power}}. \end{array}$$ (2)

Here, throughput is measured in input I/Q samples per second at the maximum operating frequency *F*_max_. Each RadioML 2016 frame contains 128 I/Q pairs, while each RadioML 2018 frame contains 1024 pairs. Dynamic power is estimated in Vivado using Switching Activity Interchange Format (SAIF) files generated from cycle-accurate simulations. Higher FoM values indicate more efficient designs, and for fair comparison all FoM values are normalized to the FINN baseline (Guo et al., 2023).

Because the proposed architecture supports configurable MMV and output-channel parallelism across different sparsity and quantization levels, the resulting design space is large. Rather than exhaustively reporting all configurations, we organize the subsequent experiments around three representative analyses. Section 4.4 isolates the impact of weight quantization on RadioML 2018, holding the architectural configuration constant; Section 4.5 then switches to RadioML 2016 to analyze how different MMV and *w*_*parallel* decompositions affect hardware cost under matched end-to-end latency; and Section 4.6 compares the proposed accelerator with prior FPGA-based AMC designs, also on RadioML 2016. Both datasets share the same SNN structure and differ mainly in input sequence length and the size of the first fully connected layer, so RadioML 2016 is used for the configuration and prior-work comparisons because its more compact configuration more clearly exposes the architectural trade-offs of interest.

### 4.4 Impact of quantization on hardware efficiency

Table 3 reports the hardware utilization, performance, and accuracy of the RadioML 2018 implementation at 16-bit, 8-bit, and 4-bit quantization, under identical *MMV* and *w*_*parallel* configurations.

**Table 3:** Resource utilization, performance, and accuracy of the RadioML 2018 implementation under different quantization precisions.

Design	#LUT	#FF	#BRAM	#DSP	Dynamic power (W)	Static power (W)	Throughput (MS/s)	Energy/S (nJ/S)	Fmax (MHz)	Latency (μs)	Accuracy (%)
2018_16bit	239,579	146,841	404	161	1.404	1.096	17.37	80.83	**138.85**	**7,270.44**	63.8
2018_8bit	**155,343**	110,915	332.5	161	0.933	1.073	17.34	53.81	138.72	7465.00	64.2
2018_4bit	164,629	**96,172**	**171**	161	**0.828**	**1.058**	**17.50**	**47.31**	137.38	7,280.68	**64.4**

All designs share the same accelerator architecture and per-layer parallelism configuration (MMV_1_ = 32, *w*_*parallel*_1_ = 2; MMV_2_ = 32, *w*_*parallel*_2_ = 4; MMV_3_ = 32, *w*_*parallel*_3_ = 4; pe_4_ = 64, simd_4_ = 16; pe_5_ = 1, simd_5_ = 2), where pe_l and simd_l denote the FINN-style processing-element and SIMD parallelism factors of the l-th fully connected layer. A uniform spatial sparsity of 50% is applied across all layers. Bold values indicate the best result in each comparison.

Under uniform 50% spatial sparsity, all three quantization levels achieve nearly identical classification accuracy, indicating that moderate-precision quantization combined with pruning is sufficient for this task. The 4-bit and 8-bit designs differ only marginally in LUT usage, since accumulation requires wider internal precision regardless of weight bit width, whereas the 16-bit design incurs noticeably higher LUT and FF usage due to its wider datapaths. BRAM consumption scales with weight precision because weights dominate on-chip memory storage, while the DSP count remains constant across all three designs since DSPs are used primarily for neuron-state decay operations that are independent of weight precision.

Throughput, *F*_max_, and latency remain largely unchanged across quantization levels because the underlying dataflow and scheduling are identical, whereas dynamic power and energy per sample clearly favor lower bit widths since reduced precision simplifies weight access and arithmetic operations.

### 4.5 Analysis of configurable design variants

Table 4 reports representative configurations of the proposed accelerator under matched end-to-end latency, isolating the effect of MMV and output-channel parallelism decomposition from differences in pipeline performance. A *balanced* configuration is one in which MMV and output-channel parallelism are jointly tuned so that per-layer latencies are aligned. Rows 2–4 are constructed as representative balanced variants by adjusting only the second convolutional layer's MMV and *w*_*parallel* while keeping their product—and hence its latency—constant. Together with the baseline of Yang et al. (2026) (Row 1) at the same sparsity, they have comparable end-to-end latency, so any difference in hardware cost, throughput, or energy can be attributed to parallelism decomposition rather than to pipeline performance.

**Table 4:** Comparison of representative configurable design variants on RadioML 2016.

Design	Q-Bit	#LUT	#FF	#BRAM	#DSP	Dynamic power (W)	Static power (W)	Throughput (MS/s)	Energy/S (nJ/S)	Fmax (MHz)	Latency (μs)	Accuracy (%)	Nor. FoM
Yang et al. (2026) 50	16	84,467	44,815	85.5	297	0.608	1.043	**23.50**	**25.87**	137.40	1,640.98	**56.29**	2.46
This work 50
16_1_16_4_16_2_2_8_1_1	16	49,155	33,358	123	51	0.746	1.051	17.36	42.97	138.85	1,827.87	**56.29**	2.54
This work 50
16_1_32_2_16_2_2_8_1_1	16	48,497	35970	140	67	0.813	1.055	19.90	40.85	**139.28**	1,807.40	**56.29**	2.71
This work 50
16_1_64_1_16_2_2_8_1_1	16	53,136	40,540	184	99	0.976	1.064	19.90	49.05	**139.28**	1,803.55	**56.29**	2.06
Yang et al. (2026) 25-20-15-20-25	16	84,808	44,969	**77**	297	0.706	1.046	**23.50**	30.04	137.40	500.82	55.54	2.11
This work 25_20_15_20_25
8_2_16_8_16_2_8_8_1_1	16	67,586	46,535	124.5	**49**	1.148	1.067	19.90	57.69	137.40	**495.40**	55.54	1.38
This work 25_20_15_20_25
8_2_16_8_16_2_8_8_1_1	8	48,321	33,966	107	**49**	0.753	1.05	19.90	37.84	138.05	497.96	55.41	2.94
This work 25_20_15_20_25
8_2_16_8_16_2_8_8_1_1	4	**42,526**	**28,726**	83	**49**	**0.541**	**1.041**	19.90	27.20	137.61	**495.40**	55.03	**4.56**

The density notation indicates per-layer sparsity: a single value denotes uniform density across all layers, and a sequence specifies layer-wise densities. For multi-line entries, the second line lists the corresponding MMV and *w*_*parallel* configuration for the five layers. Bold values indicate the best result in each comparison.

Compared with the baseline design of Yang et al. (2026), which enforces fixed output-channel parallelism and exhibits severe inter-layer latency imbalance, the proposed balanced configurations achieve latency alignment through architectural configurability rather than manual sparsity tuning. Under matched latency constraints, they reduce LUT usage to 57%–63% of the baseline while retaining 74%–85% of the throughput, demonstrating that configurable execution granularity provides a systematic, architecture-level mechanism for latency balancing in streaming SNN accelerators. Within the balanced variants themselves, increasing MMV at fixed *MMV*×*w*_*parallel* raises FF, BRAM, and DSP utilization due to higher instantaneous parallelism, while the additional control and buffering overhead also drives up LUT usage. Moderate configurations such as 32 × 2 therefore strike the best balance, achieving near-optimal throughput with substantially reduced hardware cost.

Rows 5–8 of Table 4 evaluate the same balancing mechanism under a non-uniform sparsity distribution (25-20-15-20-25), with bit width included as a secondary variable. Per-layer sparsity is fixed and configurable MMV and output-channel parallelism are tuned to match the baseline's overall inference latency (≃500μs).

Under matched latency, configurable designs consistently use fewer LUTs and DSPs than the baseline. Improved load balancing slightly raises dynamic power because average hardware utilization increases, but low-bit quantization more than compensates by reducing the per-operation power cost. The 4-bit configuration in particular uses nearly half the LUTs of the baseline while sustaining a similar throughput and incurring only a ~0.5% accuracy loss, yielding a substantially improved FoM.

### 4.6 Comparison with prior FPGA-based AMC accelerators

Table 5 compares the proposed accelerator with representative FPGA-based AMC designs on RadioML 2016.10A, including both ANN-based and SNN-based accelerators with particular attention to streaming designs. Because RadioML is a domain-specific AMC benchmark, the number of FPGA accelerators targeting this workload is limited, and these designs further differ in network model and device, so an exact match across all factors does not exist in the literature. We therefore include this broad range of designs primarily to position the proposed accelerator within the overall AMC acceleration landscape, and we make the comparison at the system level: once a comparable classification accuracy is reached, the more efficient design is the one that attains higher throughput and lower latency, power, and resource cost, regardless of the underlying model. The comparison remains fair under three conditions that hold across Table 5: all designs target the same workload and dataset; all are implemented on Xilinx FPGAs whose elementary resource units (LUTs, DSPs, and BRAMs) are structurally consistent, so that the full per-design resource breakdown can be read on a common basis; and throughput and latency are normalized to the inference time for 32 input samples (each frame contains 128 I/Q pairs), and deployment-critical performance is further summarized through a normalized FoM and an Energy/Sample metric. Within this framing, the design of Kamaleldin et al. (2026) is one of the most recent and was originally evaluated on RadioML 2018; since it is non-streaming, its latency and throughput on RadioML 2016.10A are approximately estimated from the relative input length, and we include these estimated values only as a reference point for this recent work; they are not exact and none of our architectural conclusions rely on them.

**Table 5:** Comparison of hardware resource utilization, throughput, and classification accuracy for FPGA-based AMC accelerators on RadioML 2016.

Design	Model	Platform	Q-Bit	#LUT	#FF	#BRAM	#DSP	Dynamic power (W)	Static power (W)	Throughput (MS/s)	Energy/S (nJ/S)	Fmax (MHz)	Latency (μs)	Accuracy (%)	Nor. FoM
Emad et al. (2021)	4-ANN	ZCU104	16	89,512	57,726	N/A	1,116	**0.254**	**0.593**	4.78	53.14	70	856.96	54.38	1.13
Zhang K. et al. (2023)	6-ANN	XCZU15EG	8	67,779	N/A	31	130	0.858		4.83	N/A	**200**	848.64	N/A	N/A
Wang et al. (2024)	Transformer	AXU3EG	16	20,060	**11,511**	167	115	0.6	N/A	N/A	N/A	N/A	N/A	N/A	N/A
Wang et al. (2024)	Transformer	AXU3EG	32	47,629	36,239	251	261	0.6	N/A	0.704	852.27	N/A	16,000	N/A	0.13
Rose et al. (2025)	SA-TCN	Zynq 7020	8	**16,432**	29792	**27**	48	1.7		0.03	N/A	100	377,600	N/A	N/A
Kamaleldin et al. (2026)	VGG10-ANN	XCVU9P	8	28,522	20,835	248	313	1.029	2.466	6.316	162.82	150	648.48	N/A	1.16
Maclellan et al. (2024)	4-ANN streaming	XCZU28DR	16	26,976	39,791	169	456	N/A	N/A	0.272	N/A	128	947.2	N/A	N/A
Guo et al. (2023) 50	5-SNN streaming	Virtex 709	16	66,819	47,421	63.5	118	0.921	1.054	11.45	80.44	137.40	454.85	**56.29**	1.00
Yang et al. (2026) 50	5-SNN streaming	Virtex 709	16	84,467	44815	85.5	297	0.608	1.043	23.50	**25.87**	137.40	1640.98	**56.29**	2.46
This work 50 balanced	5-SNN streaming	Virtex 709	16	48,497	35,970	140	67	0.813	1.055	19.90	40.85	139.28	1,807.40	**56.29**	**2.71**
This work 50 high throughput	5-SNN streaming	Virtex 709	16	268,331	166,244	217	105	3.67	1.182	**122.67**	29.92	138.01	**253.50**	**56.29**	0.67
This work 50 low resource	5-SNN streaming	Virtex 709	16	22,229	15,346	82.5	**15**	0.284	1.03	2.17	130.88	139.85	7,311.39	**56.29**	1.85

The density notation following each design indicates uniform sparsity across all layers. For multi-line entries, the second line summarizes the design objective—balanced: latency-balanced configuration; high-throughput: optimized for maximum throughput; low-resource: optimized for minimal hardware cost. Bold values indicate the best result in each comparison.

The ANN-based designs in Table 5 fall into three groups, each illustrating a distinct cost–efficiency trade-off. Earlier non-streaming ANN accelerators (Emad et al., 2021; Zhang K. et al., 2023; Wang et al., 2024) attain competitive accuracy but, because of dense MAC computation and the absence of inter-layer pipelining, incur high DSP cost and low throughput. More recent non-streaming designs (Rose et al., 2025; Kamaleldin et al., 2026) reduce hardware cost or raise throughput but still trail streaming architectures in latency. The streaming ANN-based design (Maclellan et al., 2024) pipelines layer execution yet is throughput-limited by its RF front-end and incurs higher DSP utilization due to higher activation precision. None of these designs combines streaming dataflow with sparse computation, which motivates the SNN-based comparison that follows.

**Figure 9:**
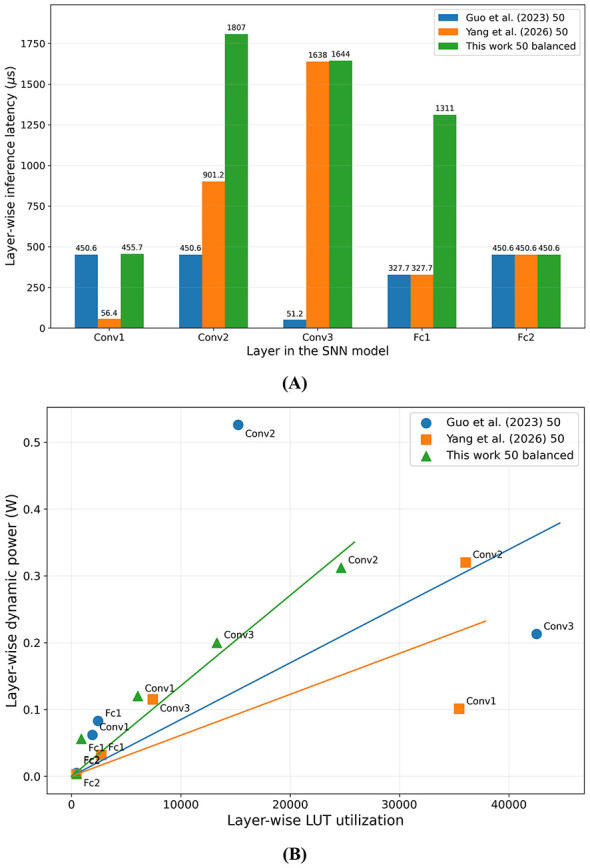
Layer-wise hardware behavior of different streaming SNN accelerator designs. **(A)** Layer-wise latency comparison. **(B)** Relationship between layer-wise LUT utilization and dynamic power consumption; layers in the proposed balanced design lie close to the fitted resource–power trend, whereas several layers in the prior designs deviate notably from it.

Streaming SNN accelerators combine event-driven computation with fully pipelined execution, making them well-suited for real-time AMC. Among them, however, the two prior streaming SNN designs in Table 5 both exhibit a coupling between parallelism and layer dimensions that constrains efficient layer-wise mapping, as confirmed by the per-layer breakdown in Table 6 and Figure 9A. In Guo et al. (2023), parallelism is bound to the number of output channels per layer: layers with many output channels (e.g., Conv3) can instantiate massive parallelism and finish quickly, whereas layers with fewer output channels (e.g., Conv1, Conv2) cannot raise their parallelism beyond the channel count and therefore retain high per-layer latency. The fast Conv3 stage must wait for slower upstream layers, so its abundant hardware does not translate into system-level latency. The design also provides no mechanism to skip zero weights, so spatial sparsity is not converted into proportional savings. In (Yang et al. 2026), parallelism is instead bound to the spatial extent of each output channel: layers with spatially large channels (e.g., Conv1, Conv2) can be made fast, but Conv3—whose channels are spatially small but numerous—cannot reduce its latency since its parallelism is capped by per-channel size. The fast Conv1 and Conv2 stages are then throttled by the slower Conv3, leaving hardware in upstream stages underutilized.

**Table 6:** Layer-wise and overall hardware cost breakdown of different streaming SNN accelerator designs.

Design	Metric	Conv1	Conv2	Conv3	Fc1	Fc2	Overall
Guo et al. (2023) 50	Power (W)	0.062	0.526	0.213	0.083	0.005	0.921
	# LUT	1,924	15,252	42,529	2,428	457	66,819
	Latency (μs)	450.56	450.56	51.20	327.68	450.56	454.85
Yang et al. (2026) 50	Power (W)	0.101	0.320	0.115	0.032	0.003	0.608
	# LUT	35,461	36,050	7,442	2,731	460	84,467
	Latency (μs)	56.38	901.18	1638.46	327.74	450.62	1,640.98
This work 50 balanced	Power (W)	0.120	0.312	0.200	0.056	0.004	0.813
	# LUT	6,061	24,648	13,302	905	460	48,497
	Latency (μs)	455.70	1,807.40	1,644.19	1,310.73	450.57	1,807.40

In both cases, the fixed coupling between parallelism and a single layer dimension produces inter-layer latency imbalance: hardware allocated to already-fast layers contributes little to end-to-end latency, since the pipeline is bounded by its slowest stage. Resolving this imbalance requires breaking the coupling itself, which is the goal of the proposed configurable architecture.

By configuring MMV and output-channel parallelism independently, the balanced configuration in Table 5 of the proposed accelerator distributes parallelism so that per-layer latencies are aligned with the bottleneck stage, attaining pipeline latency comparable to Yang et al. (2026) while using only slightly more than half of its LUT budget. Conv1 and Fc2 remain noticeably faster than the bottleneck only because their intrinsically small sizes make further latency inflation unnecessary. As reported in Table 5, this representative balanced configuration at 50% uniform weight density also reduces FF and DSP utilization at matched throughput, with the savings stemming from eliminating inter-layer latency imbalance rather than from aggressive parallelism. Figure 9B provides additional evidence from an orthogonal angle: all layers in the proposed design lie close to the fitted power–resource trend, indicating that hardware allocated to each layer is well-utilized, while several layers in the prior designs deviate notably. Dynamic power rises slightly because the better-balanced pipeline raises average hardware utilization, but the overall hardware trade-off remains favorable. These results isolate the gains attributable to configurable parallelism alone: across designs that share the same dataset, model, FPGA family, sparsity, and quantization, decoupling MMV and output-channel parallelism reduces LUT usage by 42.6% and DSP usage by 77.4% relative to the fixed-granularity baseline at matched accuracy and comparable end-to-end latency, with the savings stemming from architectural decoupling rather than from any change in sparsity, quantization, or compute primitives.

Beyond the balanced operating point, the same accelerator description can be retargeted to two additional configurations with very different optimization objectives, which are also included in Table 5 to span the performance–resource spectrum. The high-throughput configuration aggressively exploits MMV and output-channel-level parallelism to minimize inference latency, achieving 5.22 × the throughput of Yang et al. (2026) while reducing latency to 15.4% of the baseline. The low-resource configuration, in contrast, minimizes hardware cost: it requires approximately one-quarter of the LUTs and about 5% of the DSPs, enabling inference under very limited resource budgets at the price of higher per-sample energy due to MMV reconstruction overhead. Because these two configurations target fundamentally different goals, their FoM values should be interpreted in conjunction with throughput, latency, and resource utilization rather than in isolation. Together with the balanced configuration, they show that a single accelerator description can be retargeted across operating points spanning more than an order of magnitude in hardware cost, providing a practical mechanism for adapting streaming SNN deployment to diverse edge scenarios without redesigning the underlying architecture.

## 5 Discussion

Building on the experimental results above, this section discusses three implications that go beyond the specific configurations reported: the workload scope and contribution of this work, the expected scalability of the architecture to larger networks, and the limitations of the present implementation that motivate future work.

We first clarify that the experimental contribution of this paper is specifically directed at AMC. Because AMC is deployed across a wide range of edge and IoT devices whose hardware budgets differ substantially, the same trained network often needs to be retargeted across heterogeneous platforms—a need that existing streaming SNN accelerators do not address on the deployment side. The proposed architecture addresses this gap by combining sparsity-aware streaming execution with independently configurable parallelism, allowing a single trained AMC network to be efficiently retargeted across edge devices through configuration alone. The underlying mechanisms are formulated over a general convolution abstraction and could in principle be extended to other 1D conv-based SNN workloads and to higher-dimensional vision SNNs with limited algorithmic adjustments; full evaluation of such extensions is left for future work.

Although the configurations evaluated in this paper target the relatively compact SNN classifiers used in AMC, the proposed architecture is by design compatible with deeper and wider networks. This expectation follows from two well-established properties of fully pipelined streaming accelerators rather than from new experimental data. First, in such architectures the steady-state throughput is bounded by the latency of the slowest pipeline stage rather than by total network depth, so adding layers does not by itself reduce throughput as long as on-chip resources are sufficient to instantiate them (Guo et al., 2023; Yang et al., 2026). Second, end-to-end inference latency is dominated by the same bottleneck stage, since the latencies of non-bottleneck stages are absorbed by the pipeline and do not accumulate linearly with depth. The configurable parallelism introduced in this work directly targets this bottleneck stage by allowing its MMV and output-channel parallelism to be tuned independently of the rest of the network. As a result, scaling to larger networks should translate primarily into higher hardware utilization rather than into proportional latency or throughput degradation. The offline cost of producing a configuration also scales gracefully: the per-layer scheduling pipeline (channel grouping by LPT/MultiFit followed by greedy load/store ordering) is polynomial in layer size and is independent across layers, so adding more or larger layers introduces only a linear addition of inexpensive per-layer cost without combinatorial growth. Verifying this expectation on substantially larger SNN models is a worthwhile direction for future work.

Several limitations of the current implementation should also be acknowledged. First, the present design assumes offline knowledge of the weight sparsity pattern and a static network topology, which is appropriate for the inference-only AMC deployment considered here, where a trained network processes a continuous stream of varying RF inputs without runtime weight updates. Online or continual learning, in which weights are updated during operation, is not supported in the present work and is a direction we hope to explore in future work. Second, the per-layer selection of MMV and output-channel parallelism is currently performed manually, guided by the analyses in Section 4.5; an automated configuration-search procedure that takes hardware budgets and target metrics as inputs would substantially improve usability. Third, although the architecture and its supporting mechanisms are platform-agnostic in principle, all experimental results in this paper are obtained on FPGA. ASIC implementation, where the relative cost of routing, memory, and compute differs substantially, would expose new trade-offs and is left as future work.

## 6 Conclusion

This paper has presented a configurable streaming accelerator for SNNs tailored to AMC on RF signal datasets. The central goal of this work is to make three properties of an efficient streaming SNN accelerator hold at the same time: a deterministic, router-free dataflow; joint exploitation of temporal and spatial sparsity within that dataflow; and configurable parallelism that can be retargeted to heterogeneous layers and platforms. By explicitly decoupling pixel-level MMV parallelism from output-channel parallelism within a single GOAP-based streaming pipeline, the proposed architecture realizes all three properties simultaneously, generalizing our prior fixed-granularity SAOCDS design into a unified configurable framework. To make this decoupling realizable in a router-free pipeline, we developed precomputed load/store scheduling, inter-layer MMV transfer and padding mechanisms, and offline output-channel grouping strategies that exploit both spatial and temporal sparsity while flexibly adjusting performance, resource usage, and energy efficiency to meet application-specific requirements.

The proposed architecture is implemented on a Xilinx Virtex-7 FPGA and evaluated across representative configurations, quantization levels (16-bit, 8-bit, and 4-bit), and spatial sparsity conditions on the RadioML 2016 and 2018 benchmark datasets. Experimental results show that configurable parallelism achieves competitive throughput and classification accuracy with substantially reduced hardware cost compared with prior fixed-granularity streaming designs. Moreover, the same accelerator description can be retargeted across operating points spanning more than an order of magnitude in hardware cost, from high-throughput configurations that minimize inference latency to severely resource-constrained ones that minimize LUT and DSP usage.

Overall, this work shows that architectural configurability is essential for the practical deployment of streaming SNN accelerators in RF edge intelligence systems, where application requirements and hardware constraints can vary widely across deployment scenarios.

## Data Availability

The original contributions presented in the study are included in the article/supplementary material, further inquiries can be directed to the corresponding author.
